# Essential Amino Acids and Protein Synthesis: Insights into Maximizing the Muscle and Whole-Body Response to Feeding

**DOI:** 10.3390/nu12123717

**Published:** 2020-12-02

**Authors:** David D. Church, Katie R. Hirsch, Sanghee Park, Il-Young Kim, Jess A. Gwin, Stefan M. Pasiakos, Robert R. Wolfe, Arny A. Ferrando

**Affiliations:** 1 Department of Geriatrics, Donald W. Reynolds Institute on Aging, Center for Translational Research in Aging & Longevity, University of Arkansas for Medical Sciences, Little Rock, AR 72205, USA; KRHirsch@uams.edu (K.R.H.); rwolfe2@uams.edu (R.R.W.); AFerrando@uams.edu (A.A.F.); 2 Korea Mouse Metabolic Phenotyping Center, Lee Gil Ya Cancer and Diabetes Institute, College of Medicine, Gachon University, Incheon 21999, Korea; sangheepark1@gachon.ac.kr (S.P.); iykim@gachon.ac.kr (I.-Y.K.); 3 Department of Molecular Medicine, College of Medicine, Gachon University, Incheon 21999, Korea; 4 Military Nutrition Division, U.S. Army Research Institute of Environmental Medicine, Natick, MA 01760, USA; jessica.a.gwin.ctr@mail.mil (J.A.G.); stefan.m.pasiakos.civ@mail.mil (S.M.P.); 5 Oak Ridge Institute for Science and Education, Oak Ridge, TN 37830, USA

**Keywords:** protein quality, essential amino acids, protein, muscle protein synthesis, whole body protein synthesis, amino acid kinetics, nutrition, aging, anabolism

## Abstract

Ingesting protein-containing supplements and foods provides essential amino acids (EAA) necessary to increase muscle and whole-body protein synthesis (WBPS). Large variations exist in the EAA composition of supplements and foods, ranging from free-form amino acids to whole protein foods. We sought to investigate how changes in peripheral EAA after ingesting various protein and free amino acid formats altered muscle and whole-body protein synthesis. Data were compiled from four previous studies that used primed, constant infusions of L-(ring-^2^H_5_)-phenylalanine and L-(3,3-^2^H_2_)-tyrosine to determine fractional synthetic rate of muscle protein (FSR), WBPS, and circulating EAA concentrations. Stepwise regression indicated that max EAA concentration (EAAC_max_; R^2^ = 0.524, *p* < 0.001), EAAC_max_ (R^2^ = 0.341, *p* < 0.001), and change in EAA concentration (ΔEAA; R = 0.345, *p* < 0.001) were the strongest predictors for postprandial FSR, Δ (change from post absorptive to postprandial) FSR, and ΔWBPS, respectively. Within our dataset, the stepwise regression equation indicated that a 100% increase in peripheral EAA concentrations increases FSR by ~34%. Further, we observed significant (*p* < 0.05) positive (R = 0.420–0.724) correlations between the plasma EAA area under the curve above baseline, EAAC_max_, ΔEAA, and rate to EAAC_max_ to postprandial FSR, ΔFSR, and ΔWBPS. Taken together our results indicate that across a large variety of EAA/protein-containing formats and food, large increases in peripheral EAA concentrations are required to drive a robust increase in muscle and whole-body protein synthesis.

## 1. Introduction

Amino acids are the fundamental constituents of body proteins and serve as substrates for protein synthesis. Nine amino acids are considered essential amino acids (EAA), meaning they cannot be synthesized de novo, or the synthesis rate does not adequately meet the body’s demand. Therefore, EAA must be obtained through dietary protein. Dietary protein formats have a wide variance of EAA content, digestion, and absorption kinetics that can be used to meet EAA requirements [1]. 

The net balance between muscle protein synthesis (MPS) and breakdown (MPB) distinguishes the anabolic (synthesis exceeds breakdown) and the catabolic (breakdown exceeds synthesis) states. Since nonessential amino acids are normally readily available in muscle, the intracellular appearance of EAA derived from MPB or inward influx from plasma govern the anabolic response [2]. The possible fates of intracellular EAA are protein synthesis via charging the appropriate transfer ribonucleic acid (tRNA), oxidation, or efflux back to plasma. In the post-absorptive state, the primary source of intracellular EAA appearance is the accelerated rate of MPB, which is the principal determinant of the amount of intracellular EAA available as precursors for MPS [3]. Since intracellular amino acid recycling is not 100% efficient, MPB will always exceed MPS in the post-absorptive state, resulting in a net loss of muscle protein. In order to replace the lost muscle protein, exogenous EAA are required to increase circulating concentrations to induce a stimulation in MPS [4], while simultaneously reducing MPB [5,6,7]. Thus, dietary EAA are the primary stimuli for an increase in MPS and subsequent expansion of the skeletal muscle protein pool [5,6]. In addition to its role in functionality, skeletal muscle serves as a reservoir of EAA for splanchnic organs and tissue during periods of stress or insufficient dietary intake [8].

Skeletal muscle accounts for a significant portion of protein in the body, but other tissues may account for more than half the total protein turnover in the body. Recent investigations have demonstrated multiple tissues with higher turnover rates than skeletal muscle [9,10,11]. Estimates of the contribution of skeletal muscle to whole-body protein turnover range considerably (25–50%), and likely dependent upon metabolic status [12,13], and in part the tracer methodology used to quantify whole-body and MPS rates. Considering the important role of the body protein pool during catabolic stress and anabolic resistance, it is important to understand the effects of dietary EAA intakes on muscle and whole-body protein synthesis. It is also important to note that the measurement of muscle and whole-body protein synthesis are indications of protein turnover, not anabolism. Anabolism cannot be ascertained unless both protein synthesis and breakdown rates are measured. Measurement of MPB following a meal is only possible using arterial and venous catheterization, and available data using this invasive approach are limited. MPB rate can only be measured in the post-absorptive state [14,15,16,17]. Whole-body protein breakdown can be ascertained, but requires certain assumptions that are controversial [18,19,20,21]. Therefore, we chose to focus on the relationship between EAA availability and the measurement of protein synthesis according to established isotope methodology [22].

## 2. Materials and Methods 

### 2.1. Data Selection and Extraction

The present study includes data from previously published studies from our lab [23,24,25,26]. Participant demographics and treatments are outlined in Table 1. Written informed consent was obtained from all subjects, and studies were approved by the Institutional Review Board (#89220, 205336, 206579, 206814, and 217658) at the University of Arkansas for Medical Sciences. Participants were healthy young or older males who had refrained from physical activity for at least 72-h. Studies were conducted after an overnight fast, and isotope infusion was utilized to determine mixed MPS and whole-body protein synthesis. 

### 2.2. Infusion Trials

Studies were conducted after an overnight fast, and isotope infusion was utilized to determine mixed MPS and whole-body protein synthesis. Two intravenous catheters were placed; one in the antecubital space for the continuous isotope infusions, and the second in the contralateral dorsal hand vein for serial blood draws. The dorsal hand vein was kept warm using heating pads to reflect arterialized blood [27]. After collecting the baseline blood sample, primed, constant infusions of L-(ring-^2^H_5_)-phenylalanine and L-(3,3-^2^H_2_)-tyrosine were started and maintained. A priming dose of L-(ring-^2^H_4_)-tyrosine was also administered at the same time to achieve isotopic equilibrium of L-(ring-^2^H_4_) tyrosine enrichment derived from L-(ring-^2^H_5_)-phenylalanine for the measurement of phenylalanine hydroxylation. All isotopes were purchased from Cambridge Isotope Laboratories (Andover, MA, USA) and the preparations were constituted by the research pharmacist at UAMS.

### 2.3. Analytic Procedures

Plasma was precipitated with 125 µL of 10% sulfosalicylic acid (SSA), centrifuged, and the supernatant was used to determine EAA concentrations using the internal standard technique [28]. Phenylalanine and tyrosine enrichments were measured using the tert-butyldimethylsilyl derivative and gas chromatography-mass spectrometry. Ions of mass-to-charge ratio of 234, 235, and 239 for phenylalanine and of 466, 467, 468, and 470 for tyrosine were monitored with electron impact ionization and selective ion monitoring. Serum insulin concentrations were measured using a Siemens Immulite 2000 XPi (Siemens Medical Solutions USA, Inc., Malvern, PA, USA).

Muscle samples were weighed, and tissue proteins were precipitated with 0.5 mL of 4% SSA. Samples were then homogenized, centrifuged, and the muscle pellet (bound protein) was washed, dried, and hydrolyzed in 0.5 mL of 6 N HCl at 105 °C for 24-h. Mixed muscle-bound protein enrichments were determined as described above for plasma enrichments.

### 2.4. Muscle Fractional Synthetic Rate

The precursor-product model was used to determine mixed MPS (i.e., fractional synthetic rate) [29]:

Mixed MPS (%/h) = ((E_BP2_ − E_BP1_)/(E_p_)) × 60 × 100, (1)

where E_BP1_ and E_BP2_ are the enrichments of bound L-(ring-^2^H_5_)-phenylalanine in muscle collected in the postabsorptive and postprandial states. In the postprandial state, the precursor enrichment (Ep) is the calculated area under the curve (AUC) for L-(ring-^2^H_5_)-phenylalanine enrichment in the plasma since it more accurately reflects the blood perturbations consistent with our and others’ previous work [23,24,25,30,31]. Factors 60 and 100 were used to express mixed MPS as percent per hour. Both the postprandial FSR and change in FSR (ΔFSR) were used for statistical analysis.

### 2.5. Whole-Body Protein Synthesis

Whole-body protein synthesis rates were calculated based on the determinations of the rate of appearance (R_a_) into the plasma of phenylalanine and tyrosine and the fractional Ra of endogenous tyrosine converted from phenylalanine [22]. The phenylalanine (Phe) and tyrosine (Tyr) plasma enrichment mean and AUC were calculated for the postabsorptive and postprandial states, respectively. Whole-body protein synthesis was calculated by dividing kinetic values of phenylalanine by its fractional contribution to protein. The following equations were used to calculate whole-body protein synthesis [23,25,30]:

Total plasma R_a_Phe = F/E, (2)

Fractional R_a_ of Tyr from Phe = E_Tyr M+4_/E_Phe M+5_, (3)

Phe hydroxylation = fractional R_a_ of Tyr from Phe × R_a_ Tyr, (4)

PS = ((R_a_ Phe − Phe hydroxylation) × 25), (5)

where E is enrichment of respective tracers at plateau and expressed as mole percent excess (MPE), calculated as TTR/(TTR + 1). F is the respective tracer infusion rate into a venous side. E_Tyr M+4_ and E_Phe M+5_ are plasma enrichments of tyrosine and phenylalanine tracers at M + 4 and M + 5 relative to M + 0, respectively. In the fed state, fractional R_a_ of Tyr from Phe was divided by 0.8 to account for hepatic dilution [32]. The correction factor of 25 is for conversion of phenylalanine values to total protein based on the assumption that the contribution of phenylalanine to skeletal muscle protein is 4% (100/4 = 25) [3]. Phe hydroxylation is the R_a_ of tyrosine derived via endogenous hydroxylation of phenylalanine. Post-absorptive whole-body protein synthesis was subtracted from the postprandial whole-body protein synthesis and expressed as grams per hour. 

### 2.6. Amino Acid Pharmacokinetics

The temporal response of the EAA curve was used to calculate the following variables for statistical analysis: Area under the curve above baseline (AUCi), max EAA concentration (EAAC_max_), EAA concentration change from baseline to EAAC_max_ (ΔEAA), and the rate to EAAC_max_. 

### 2.7. Statistical Analysis

To identify the best EAA predictor of ΔFSR, postprandial FSR, and ΔWBPS a stepwise linear regression model was used to assess the contribution of independent (AUCi, EAAC_max_, ΔEAA, rate to EAAC_Max_, age, lean body mass, total kilocalories, total carbohydrates, total protein, total fat, and protein quality (EAA:total protein)) variables. Relationships between variables were assessed with Pearson’s product moment correlation. A priori α < 0.05 was used and data are expressed as means ± SEMs. Statistical analyses were performed with IBM SPSS software (version 26; IBM Corp. Armonk, NY, USA). 

## 3. Results

### 3.1. Stepwise Regression

The best predictors for ΔFSR, postprandial FSR, and ΔWBPS are presented in Table 2**.** Stepwise regression indicated that EAAC_max_ (R^2^ = 0.341, *p* < 0.001), EAAC_max_ (R^2^ = 0.524, *p* < 0.001), and ΔEAA (R^2^ = 0.345, *p* < 0.001) were the strongest predictors for ΔFSR, postprandial FSR, and ΔWBPS, respectively. 

### 3.2. Correlations

**Figure 1:**
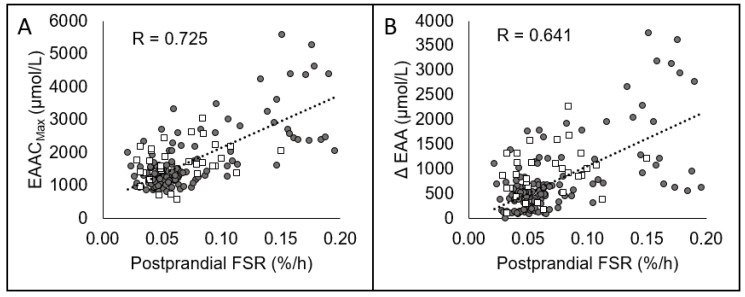
Relationships between postprandial fractional synthetic rate (FSR) to maximum essential amino acid (EAA) concentration (EAAC_max_; Panel **A**) and the change in EAA concentrations from baseline to EAAC_max_ (ΔEAA; Panel **B**). Individual participant data from our lab in filled circles (●), means gathered from post-feeding data from other studies from the literature [6,27,29,30,31,32,33,34,35,36,37,38,39] in open boxes (□). Means were not used in statistical analysis, they are used to represent consistency in data relationships between labs.

The relationships between postprandial FSR and EAAC_max_ and ΔEAA are highlighted in Figure 1A,B. Correlations between macronutrients and protein kinetics are outlined in Table 3. Correlations between AUCi, EAAC_max_, ΔEAA, and rate to EAAC_max_ and postprandial FSR, ΔFSR, and ΔWBPS are outlined in Table 4. 

**Figure 2:**
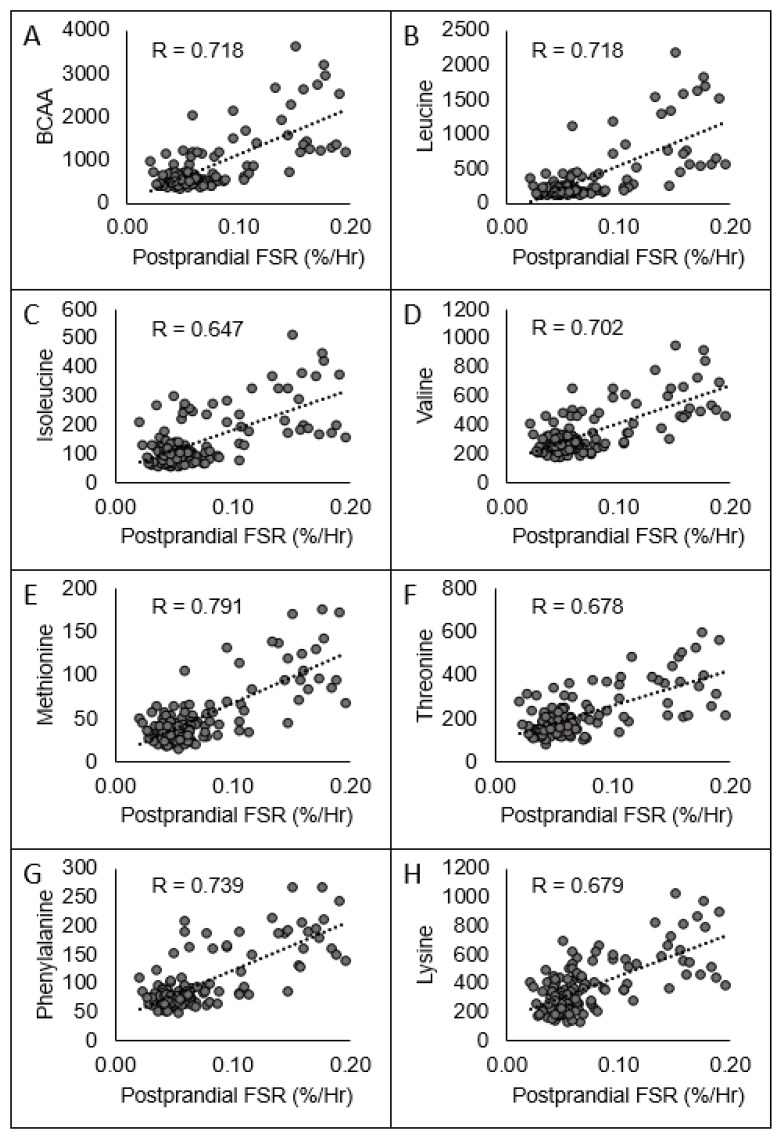
Relationships between C_Max_ (umol/L) of the sum of the branched chain amino acids (**A**; BCAAs) and individual amino acids (**B**–**H**) to postprandial FSR. Not pictured: non-significant relationships between tryptophan and histidine.

The C_max_ values for the sum of the branched chain amino acids (BCAA) and all individual EAA, highlighted in Figure 2A–H, were significantly (*p* ≤ 0.05) correlated to the postprandial FSR (r = 0.647–0.761), with the exception of tryptophan and histidine; ΔFSR (r = 0.545–0.621), again with the exception of tryptophan and histidine; and ΔWBPS (r = 0.439–0.541), again with the exception of tryptophan and histidine. The AUCi for the sum of the BCAA and all individual EAA were significantly (*p* ≤ 0.05) correlated to the postprandial FSR (r = 0.322–0.690), except for tryptophan and histidine; ΔFSR (r = 0.331–0.528), except tryptophan and histidine, and ΔWBPS (r = 0.307–0.590) except tryptophan and histidine. The Δ for the sum of the BCAA and all individual EAA were all significantly (*p* ≤ 0.05) correlated to the postprandial FSR (r = 0.197–0.701), except for histidine, ΔFSR (r = 0.481–0.567) except tryptophan and histidine, and ΔWBPS (r = 0.218–0.462) except tryptophan.

## 4. Discussion

Our results indicate that greater peripheral EAA concentrations are related to the stimulation of MPS and WBPS and in response to feeding. Our stepwise regression results indicated that the strongest predictors for postprandial FSR, ΔFSR, and ΔWBPS were measures of EAA concentrations; explaining approximately 30–50% of the variance in protein synthesis measures. The results support the requirement for increased peripheral EAA concentrations to stimulate MPS and WBPS. Expressed differently, a larger EAA gradient between the extracellular (peripheral) and intracellular compartments enables greater inward transport and subsequent charging of tRNA and stimulation of synthesis [3,25].

Based upon the average basal EAA concentrations (961 µmol/L) in the current dataset, the regression equation indicates that a 100% increase in EAA concentrations (EAAC_Max_ = 1922 µmol/L) would result in a ΔFSR of 0.020, or a ~34% increase (based upon the average post-absorptive FSR; 0.059). Our results are nearly identical to those of Pennings and colleagues [33] who demonstrated a significant correlation (r = 0.55, *p* < 0.01) between ΔEAA concentrations and postprandial FSR following the ingestion of whey, casein, or casein hydrolysate protein in older men. Considering the large variance of FSR measures [34], we feel the similar correlation coefficients (r = 0.64 and r = 0.55) obtained by separate laboratories provides consistent evidence for an established relationship between peripheral EAA concentrations and FSR within the range of protein consumed. Further, study means collected from the literature [7,33,35,36,37,38,39,40,41,42,43,44,45] fall in line with measured values from participants in our laboratory (Figure 2). Previous reports have demonstrated that the stimulation of the inward amino acid transport is the mechanism by which MPS is stimulated [46]. For example, Bohé and colleagues [4] demonstrated MPS was related to extracellular, not intracellular, EAA concentrations during infusion of mixed amino acids at four different rates. This seminal work provides the mechanistic foundation of the control of MPS by peripheral EAA. However, infusion of mixed amino acids directly into blood bypasses first pass splanchnic uptake and does not accurately represent a real-life feeding situation. Nonetheless, studies have routinely demonstrated a dose-response relationship between total protein intake and FSR throughout a wide range of EAA sources such as; free-form EAA, whey, egg, soy, beef [31,37,47,48,49,50,51,52]. Although EAA concentrations were not always measured, a reasonable assumption can be made that increasing the dose of EAA source results in higher peripheral EAA concentrations [23,30,37,39,53]. The oral ingestion studies indicate a point where increasing doses of complete protein, and thus EAA concentrations, no longer further stimulate FSR. We did not observe this in our data since the maximal EAA dose was 11.2 g and most likely below a dose (~15 g of EAA), which achieves a maximal FSR response [5,7,37,47,52,54,55]. Altogether, these data demonstrate large increases in peripheral/extracellular EAA concentrations are an important factor in driving the postprandial rise in FSR. 

In addition to a robust sample size, a strength of the present analysis is the measurement of all EAA concentrations, allowing for correlations between the sum of the EAA, BCAA, and each individual EAA on its own. Many studies only measure leucine or the BCAAs. Leucine has been shown to be an important amino acid within a protein source and to serve as a “trigger” to stimulate MPS via mammalian target or rapamycin complex 1 signaling [56]. Our data are consistent with these findings in that we observed significant relationships between leucine concentrations and both postprandial FSR and ΔFSR. More importantly, we observed significant correlations between all the individual EAA, with the exception of tryptophan and histidine, and FSR measures. Previous work has demonstrated the requirement for all the combined EAA to stimulate MPS [57]. Numerous studies have demonstrated the inability of leucine and BCAAs alone to induce a rise in MPS or improvements in muscle hypertrophy or strength with chronic use [57,58,59,60,61]. Partial EAA administration (BCAA or leucine) produces a transient anabolic response, at best, due to the activation of translational molecular pathways [62]. Unless an adequate supply of EAA are available, MPB must increase to compensate for the lack of precursors. For this reason, the correlation of nearly all the EAA with FSR denotes the adequacy of precursor provision. 

Our results demonstrate that ΔWBPS was also significantly correlated with EAA AUCi, EAAC_max_, ΔEAA, and rate to EAAC_max_, with ΔEAA being the strongest predictor of ΔWBPS (Table 2). To our knowledge, we are the first to demonstrate significant relationships between peripheral EAA concentrations and ΔWBPS. The lack of investigation in this area makes direct comparisons difficult and indicates an important gap in the literature. Measurement of WBPS provides additional insight beyond MPS. Though skeletal muscle constitutes the majority of the body protein pool, the labile splanchnic tissue contributes substantially to whole-body protein turnover [9,10,11,63]. Recent work provides evidence of disparate muscle and whole-body anabolism at different protein intakes [30,52,64,65]. Thus, nutritional recommendations based upon muscle response alone fails to consider the beneficial whole-body effects of elevated EAA concentrations. Similar to results concerning FSR, studies demonstrating higher WBPS in response to increased amounts of free-form EAA, intact protein, and food sources are in agreement with our results [30,39,52,64,65]. Furthermore, the absorption and digestion of an EAA source are important in determining EAA availability and whole-body protein metabolism [66]. Our data are consistent with these findings, as total energy, and protein within an EAA source was negatively correlated, while the ratio of EAA:total protein was positively correlated with postprandial FSR, ΔFSR, and ΔWBPS. The large digestive requirement of whole food protein sources, as compared to simpler EAA matrices, results in a slower liberation and subsequent entry of EAA into circulation. 

Our results provide evidence for peripheral EAA concentrations dictating protein synthesis at both the muscle and whole-body level. An attenuated rate of EAA liberation from digestion results in lower peripheral EAA concentrations at the membrane receptor, reducing the EAA concentration gradient from plasma to intracellular space. Transmembrane transport of amino acids is the determinant of the availability of circulating EAA for protein synthesis [67], and maximal transport rates occur when EAA gradients between intra- and extracellular concentrations are large. By maximizing the EAA gradient, the rate limiting step is no longer the supply of substrate (i.e., precursor amino acids) but rather the quantity of translational machinery (i.e., tRNA and ribosome content). This notion is supported by our previous work in rabbits demonstrating liver, with protein synthesis rates ~4-fold greater than muscle, corresponded with 4-fold greater tRNA content than muscle [68]. These data indicate that achieving a large EAA gradient is an important determinate of the protein synthetic response to feeding. 

The current analysis is not without limitations. First, the analysis was accomplished by combining data from multiple studies, rather than a single study systematically altering protein formats and amounts. While performing a single study would be ideal, the present analysis is arguably only plausible by leveraging multiple studies. Further, the variety of interventions resulted in varied postprandial EAA responses in a large group of human subjects. As a result, our analysis clearly demonstrates the importance elevated EAA concentrations have on FSR and WBPS measurements. Another limitation is that we only measured one side of total protein status at both the muscle and whole-body level via protein synthesis. Thus, our results should be interpreted as indications of protein turnover, not protein anabolism. Measurement of only one variable in the protein balance equation potentially explains the low(er) variance in muscle and WBPS related to increased peripheral EAA concentrations. Our dataset has many cases where the modest rise in peripheral EAA would require a concomitant increase in MPB to supply EAA precursors for muscle and WBPS. At the muscle level it is often asserted that rises in MPS dictate the anabolic response, and subsequent muscle hypertrophy. However, this assertion can lead to incorrect conclusions. For example, burn injury patients display a 2-fold increase in FSR values; however, MPB increases by 3-fold, such that muscle protein balance is dramatically negative [69]. This also indicates that EAA availability, whether from ingestion or endogenous sources is the primary determinate of protein synthesis. While not included in the present analysis, data from our lab indicated that whole-body protein breakdown is strongly correlated with WBPS in the postprandial period. Taken together it is likely that the measurement of circulating EAA concentrations would be more predictive of anabolism if protein breakdown was also determined. 

## 5. Conclusions

This work extends previous findings denoting the relationship between peripheral EAA concentrations and FSR by outlining responses to various formats of EAA intake. Our regression equations indicate that a 100% increase in peripheral EAA concentrations results in approximately a 34% increase in FSR from post-absorptive conditions. The present analysis expands upon previous findings by demonstrating that this relationship also extends to whole-body protein synthesis over a wide range of EAA sources. These findings also demonstrate that ingestion formats with a high proportion of EAA relative to total protein content represent a viable means of improving muscle and whole-body protein synthetic responses. Taken together, EAA sources that produce a large and rapid increase in peripheral EAA concentrations are recommended to improve muscle and whole-body protein synthesis. 

## Figures and Tables

**Table 1:** Participant demographics from compiled studies ^1.^

Study	Group	N (M/F)	Age (y)	BM (kg)	BMI (kg/m^2^)	LBM (kg)	BF (%)
**Park 2020 *European Journal of Nutrition* [25]**	3.5 oz Ground Beef and 2 green kiwis	11 (5/5)	72.5 ± 1.9	82.5 ± 2.2	28.7 ± 0.8	48.5 ± 2.5	36.4 ± 2.1
	3.5 oz Ground Beef and 2 gold kiwis						
**Park 2020 *Journal of International Society of Sports Nutrition* [23]**	2.4 g Whey + 3.2 g Free Form EAA	8 (3/5)	21.4 ± 0.5	73.8 ± 4.8	24.6 ± 0.8	51.6 ± 4.9	21.1 ± 2.2
	4.8 g Whey + 6.4 g Free Form EAA						
	12.6 g Whey	8 (4/4)	26.9 ± 2.0	76.2 ± 3.1	25.7 ± 1.6	49.5 ± 2.6	24.8 ± 4.1
**Park 2020 *Journal of Nutrition* [26]**	2 oz Ground Beef	8 (4/4)	21.8 ± 2.2	76.3 ± 4.6	24.9 ± 1.0	49.2 ± 4.1	30.1 ± 3.1
	2 oz Beef Sirloin	8 (4/4)	23.9 ± 1.6	68.0 ± 4.0	23.5 ± 1.0	43.5 ± 3.3	31.0 ± 2.4
	2 Cooked Eggs	8 (4/4)	23.9 ± 1.9	74.2 ± 5.4	24.4 ± 1.3	49.1 ± 3.4	27.5 ± 2.5
	2 oz Pork Loin	8 (4/4)	22.1 ± 1.0	74.3 ± 3.5	24.5 ± 0.9	51.8 ± 4.6	29.9 ± 3.9
	1/2C Kidney Beans	8 (4/4)	23.8 ± 1.9	69.8 ± 5.7	24.1 ± 1.7	44.8 ± 3.1	30.1 ± 2.5
	2T Peanut Butter	8 (4/4)	20.3 ± 1.5	70.4 ± 3.8	24.1 ± 1.1	48.1 ± 3.8	27.1 ± 2.3
	4 oz Tofu	8 (4/4)	25.9 ± 2.2	75.9 ± 2.2	25.9 ± 1.0	49.7 ± 4.2	33.0 ± 3.0
	1 oz Mixed Nuts	8 (4/4)	24.3 ± 2.1	74.3 ± 5.3	24.9 ± 1.2	49.3 ± 3.9	32.2 ± 2.2
**Church 2020 *Journal of International Society of Sports Nutrition* [24]**	3.6 g Free Form EAA	11 (5/6)	68.8 ± 1.8	81.4 ± 5.69	31.8 ± 5.7	49.7 ± 3.6	35.7 ± 2.2
	10.8 g Free Form EAA	12 (8/4)	67.4 ± 1.5	83.4 ± 5.5	27.5 ± 1.3	53.1 ± 3.9	35.4 ± 3.2
**Sample Means**		-	39.9 ± 2.0	76.1 ± 1.2	26.1 ± 0.6	49.5 ± 0.9	32.2 ± 0.8

^1^ Data reported as mean ± SEM. BM = body mass; BMI= body mass index; LBM = lean body mass; BF = body fat; y = years; kg = kilograms; m = meters; oz. = ounces; g = grams; EAA = essential amino acids; C = cup; T = tablespoon. Data are means ± SEM.

**Table 2:** Stepwise regression results ^1^.

Best Predictor	R^2^	*p*	Formula
**ΔFSR**			
EAAC_Max_	0.341	≤0.001	(0.00001933 × EAAC_Max_) − 0.017
**Postprandial FSR**			
EAAC_Max_	0.524	≤0.001	(0.00003307 × EAAC_Max_) + 0.016
**ΔWBPS**			
ΔEAA	0.345	≤0.001	(0.001 × ΔEAA) + 0.242

^1^ ΔFSR = postprandial fractional synthetic rate minus postabsorptive FSR; ΔWBPS = postprandial whole-body protein synthesis minus postabsorptive whole-body protein synthesis; EAA = essential amino acid; C_max_ = max concentration; Δ = change in concentration from baseline to C_Max_.

**Table 3:** Correlations between macronutrients and physiological variables ^1^.

		kcals	Protein (g)	EAA (g)	CHO (g)	Fat (g)	EAA:Protein
Postprandial FSR (%/h)	R	−0.334 *	−0.189 *	0.230 *	−0.210 *	−0.402 *	0.644 *
	N	134	134	134	134	134	134
ΔFSR (%/h)	R	−0.346 *	−0.205 *	0.227 *	−0.255 *	−0.383 *	0.585 *
	N	134	134	134	134	134	134
ΔWBPS (g/h)	R	−0.227 *	0.033	0.386 *	−0.269 *	−0.279 *	0.409 *
	N	112	112	112	112	112	112
EAA AUCi (umol/L/min)	R	0.096	0.400 *	0.822 *	0.063	−0.121	0.379 *
	N	134	134	134	134	134	134
EAAC_max_ (umol/L)	R	−0.429 *	−0.169	0.510 *	−0.342 *	−0.524 *	0.781 *
	N	134	134	134	134	134	134
Δ[EAA] (umol/L)	R	−0.383 *	−0.094	0.604 *	−0.323 *	−0.495 *	0.733 *
	N	134	134	134	134	134	134
Rate to Peak EAAC_max_ (umol/L/min)	R	−0.546 *	−0.303 *	0.418 *	−0.430 *	−0.611 *	0.841 *
	N	134	134	134	134	134	134

^1^ kcal = kilocalories; EAA = essential amino acid; CHO = carbohydrate; EAA: protein = EAA to total protein ratio; g = grams; h = hours; FSR = fractional synthetic rate; ΔFSR = postprandial FSR minus postabsorptive FSR; WBPS = whole-body protein synthesis; ΔWBPS = postprandial WBPS minus postabsorptive WBPS; EAA AUCi = EAA area under the curve above basal; EAAC_max_ = max EAA concentration; ΔEAA = EAAC_max_ minus basal EAA concentration. * = significant correlation.

**Table 4:** Correlations between protein kinetics and essential amino acid (EAA) pharmacokinetics ^1^.

		Postprandial FSR (%/h)	ΔFSR (%/h)	ΔWBPS (g/h)
EAA AUCi (umol/L/min)	R	0.475 *	0.420 *	0.438 *
	N	130	130	112
EAACmax (umol/L)	R	0.724 *	0.584 *	0.559 *
	N	130	130	112
Δ[EAA] (umol/L)	R	0.641 *	0.562 *	0.587 *
	N	130	130	112
Rate to Peak EAACmax (umol/L/min)	R	0.626 *	0.577 *	0.444 *
	N	130	130	112

^1^ FSR = fractional synthetic rate; h = hours; ΔFSR = postprandial FSR minus postabsorptive FSR; WBPS = whole-body protein synthesis; ΔWBPS = postprandial WBPS minus postabsorptive WBPS; EAA AUCi = EAA area under the curve above basal; EAAC_max_ = max EAA concentration; ΔEAA = EAAC_max_ minus basal EAA concentration. * = significant correlation.
